# miRNA‐384‐3p alleviates sevoflurane‐induced nerve injury by inhibiting Aak1 kinase in neonatal rats

**DOI:** 10.1002/brb3.2556

**Published:** 2022-06-20

**Authors:** Yuanyuan Chen, Xuan Gao, Hao Pei

**Affiliations:** ^1^ Department of Anesthesiology Yancheng Maternity and Child Health Care Hospital Yancheng Jiangsu China; ^2^ Department of Anesthesiology Shanghai Blue Cross Brain Hospital Shanghai China; ^3^ Department of Anesthesiology Children's Hospital of Fudan University Shanghai China

**Keywords:** Aak1, miRNA‐384‐3p, nerve injury, sevoflurane

## Abstract

**Objective:**

Sevoflurane is a common anesthetic and is widely used in pediatric clinical surgery to induce and maintain anesthesia through inhalation. Increasing studies have revealed that sevoflurane has neurotoxic effects on neurons, apoptosis, and memory impairment. miR‐384 is involved in the process of neurological diseases. However, the role of miRNA‐384‐3p in sevoflurane‐induced nerve injury is not clear. This study focused on exploring the roles and mechanisms of miRNA‐384‐3p in sevoflurane‐induced nerve injury.

**Methods:**

Seven‐day‐old rats were exposed to 2.3% sevoflurane to induce nerve injury. The morphological changes in neurons in the hippocampal CA1 region were detected by HE staining and Nissl staining. Neuronal apoptosis was detected by TUNEL and Western blot assays. Spatial memory and learning ability were detected by the Morris water maze assay. The target gene of miRNA‐384‐3p was verified through a luciferase reporter assay. A rescue experiment was used to confirm the miRNA‐384‐3p pathway in sevoflurane‐induced nerve injury.

**Results:**

Sevoflurane reduced miRNA‐384‐3p expression in the rat hippocampus. miRNA‐384‐3p alleviated sevoflurane‐induced morphological changes in hippocampal neurons and apoptosis of neurons in the hippocampal CA1 region. Meanwhile, miRNA‐384‐3p attenuated the decline in spatial memory and learning ability induced by sevoflurane. miRNA‐384‐3p alleviated sevoflurane‐induced nerve injury by inhibiting the expression of adaptor‐associated kinase 1 (Aak1).

**Conclusion:**

Our findings revealed the role and mechanism of miRNA‐384‐3p in sevoflurane‐induced nerve injury, suggesting that miRNA‐384‐3p could be a novel and promising strategy for reducing sevoflurane‐induced neurotoxicity.

## Graphical abstract

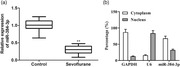

## 1 INTRODUCTION

Sevoflurane, an inhalation anesthetic, is widely used in clinical surgical operations (Egan, 2015; Yi et al., 2015). However, recent studies have shown that sevoflurane has neurotoxic effects, increases neuronal apoptosis, and reduces learning and memory ability (H. He et al., 2018; Perez‐Zoghbi et al., 2017). The mechanism of sevoflurane‐induced neurotoxicity remains mostly unknown. Hence, it is necessary to explore the underlying molecular mechanism of sevoflurane‐induced neurotoxicity to reduce sevoflurane‐induced nerve injury.

MicroRNAs (miRNAs) are noncoding RNAs, and their expression is involved in various physiological and pathological processes (Gjorgjieva et al., 2019; Sun et al., 2018). Some studies have confirmed that miRNAs play a vital role in sevoflurane‐induced neurotoxicity. For example, Zhao et al. (2018) found that sevoflurane upregulates miR‐34a expression in the hippocampus. miR‐34a also promoted neuronal apoptosis and memory impairment induced by sevoflurane through the wnt1/β‐catenin pathway (Zhao et al., 2018). In neonatal rats, the level of miR‐96 is positively correlated with the concentration of exposed sevoflurane. The increased expression of miR‐96 aggravates sevoflurane‐induced hippocampal neuron apoptosis and cognitive function injury (C. Xu et al., 2019). X. He et al. (2007) found that miR‐384 expression was higher in the hippocampus than in other tissues. In addition, miR‐384‐5p expression was more than 10 times higher than that of miRNA‐384‐3p in the rat hippocampus (X. He et al., 2007). Liu et al. found that chronic cerebral ischemia increased miR‐384 expression in the hippocampus and hippocampal neurons. Knockdown of miR‐384 inhibits the apoptosis of hippocampal neurons induced by chronic cerebral ischemia (Liu et al., 2019). Similarly, miR‐384‐5p promotes neurotoxicity and attenuates learning and memory in rats (Jiang et al., 2016; Q. Xu et al., 2019). However, whether the roles of miRNA‐384‐3p are consistent with those of miR‐384‐5p in neurotoxicity remains obscure. Therefore, we focused on exploring the effects of miRNA‐384‐3p on sevoflurane‐induced neurotoxicity.

Aak1, adaptor‐associated kinase 1, has been reported to be associated with nervous‐related diseases and nerve injury (Shi et al., 2014). For instance, Aak1 regulates clathrin‐mediated endocytosis, thereby affecting the cognitive ability of AD mice (Fu et al., 2018). However, the role of Aak1 in anesthesia‐induced neurotoxicity remains unclear.

The role and mechanism of miRNA‐384‐3p in sevoflurane‐induced neurotoxicity were investigated in this study, and the results confirmed that miRNA‐384‐3p attenuated sevoflurane‐induced neuronal apoptosis and memory disorder by inhibiting the expression of Aak1. Our findings suggest that miRNA‐384‐3p may be a promising strategy for resolving sevoflurane‐induced nerve injury during clinical surgery.

## 2 MATERIALS AND METHODS

### 2.1 Animals and treatment

Seven‐day‐old Sprague–Dawley rats were used in this study. They were obtained from the GemPharmatech Company (Nanjing, China). All rats were kept on a 12‐h light–dark cycle in individually ventilated cages at 21 ± 1°C with free access to food and water. All animal experiments were approved by the Institutional Animal Care and Use Committees and performed according to the institution's guidelines and animal research principles.

The rats were randomly divided into three groups: control, sevoflurane, and miRNA‐384‐3p agomir injection. Each group consisted of 12 rats. Sevoflurane was used to anesthetize rats as previously described (Zhou et al., 2017). Briefly, rats were exposed to 2.3% sevoflurane for 2 h every day for 3 continuous days. The gas flow was 2 L/min, and the concentration of sevoflurane was measured by a gas monitor (Detex Ohmeda, CO, USA). The NPS‐A3 heating device (Midea Group, Beijiao, China) was used to heat the chamber up to 38°C. Rats in the sevoflurane group were injected with 2 nmol agomir NC (volume is 2 μl) into the hippocampus on the left lateral cerebral ventricles after the first day of exposure to sevoflurane. The miRNA‐384‐3p agomir was purchased from RiboBio (Guangzhou, China) and diluted with Entranster transfection reagent (Engreen Biosystem Co., Beijing, China). Then, bilateral intrahippocampal administration was performed by injection with 2 nmol miRNA‐384‐3p agomir (volume is 2 μl) into the hippocampus using a stereotaxic apparatus (RWD Life Science, Shenzhen, China) and a 33‐gauge beveled NanoFil needle. On the first day of exposure to sevoflurane, the cells were exposed to sevoflurane for 2 days. Control group rats were exposed to air for 2 h/day and over 3 consecutive days. After being exposed to sevoflurane for 3 days, the rats were euthanized, and the hippocampus was collected for further experiments.

### 2.2 Cell isolation and culture

The hippocampus was dissected from neonatal rats (7 days old), triturated, and dissociated through trypsin. The dissociated cells were filtered and centrifuged and then resuspended in Dulbecco's Modified Eagle Medium/F12 medium (DMEM/F12, Thermo‐Scientific, MA, USA). Then, the cells were seeded onto dishes coated with poly‐D‐lysine and cultured with DMEM/F12 supplemented with 10% fetal bovine serum (FBS, Thermo Scientific, MA, USA), 1% glutamine, 4.5 g/L B27 plus glucose, and 1% penicillin–streptomycin (Sigma–Aldrich, MI, USA). After culturing for 3 days, 5 μg/ml cytosine arabinoside C (Sigma–Aldrich, MI, USA) was added to the medium and cultured for 24 h. The neurons were cultured in a humidified incubator at 37°C and 5% CO_2_ for 14 days.

### 2.3 Cell treatment and transfection

Neurons were cultured in a humidified incubator chamber with a gas mixture of 1% sevoflurane, 94% air and 5% CO_2_ for 6 h. Sevoflurane was delivered to the chamber at a rate of 10 L/min through a vaporizer (Datex‐Ohmeda, Helsinki, Finland). Control neurons were cultured in a humidified incubator with 95% air and 5% CO_2_.

Hippocampal neurons, including control and sevoflurane‐exposed neurons, were seeded into 24‐well plates at 10^4^ cells/well. When the confluence of the cells reached 60%, Lipofectamine 3000 (Thermo Scientific, MA, USA) was used for transfection. miRNA‐384‐3p mimics, NC mimics, pcDNA‐Aak1 vector (pc‐Aak1), and pcDNA control vector (pc‐NC) were obtained from GeneChem (Shanghai, China). miRNA‐384‐3p mimics and NC mimics were transfected into control neurons. NC mimics and pc‐NC were transfected into control neurons simultaneously. NC mimics and pc‐NC, miRNA‐384‐3p mimics and pc‐NC, and miRNA‐384‐3p mimics and pc‐Aak1 were transfected into sevoflurane‐exposed neurons simultaneously. The transfection concentration was 10 nM. After transfection for 48 h, the cells were collected for further experiments.

### 2.4 Real‐time quantitative polymerase chain reaction

Total RNA was isolated from hippocampal tissue or transfected neurons by using TRIzol reagent (Thermo‐Scientific, MA, USA). RNA was reverse transcribed into cDNA by using the PrimeScript RT reagent kit (Takara, Japan). A SYBR green PCR kit (Vazyme, Nanjing, China) was used to perform real‐time quantitative polymerase chain reaction (RT–qPCR). U6 and GAPDH were used to normalize the relative expression of miRNA‐384‐3p and Aak1. The miRNA‐384‐3p forward primer sequence (5′−3′) was AATTCCTAGAAATTGTT, and the reverse primer sequence (5′−3′) was AGTGCAGGGTCCGAGGTATT. The U6 forward primer sequence (5′−3′) was CTCGCTTCGGCAGCACATATACT, and the reverse primer sequence (5′−3′) was ACGCTTCACGAATTTGCGTGTC. The Aak1 forward primer sequence (5′−3′) was CGGGTCACTTCCGGGTTTA, and the reverse primer sequence (5′−3′) was TTCTTCTCCGGTTTCAGCCC. The GAPDH forward primer sequence (5′−3′) was GAACGGGAAGCTCACTGG, and the reverse primer sequence (5′−3′) was GCCTGCTTCACCACCTTCT.

### 2.5 Subcellular fractionation

After hippocampal microdissection, tissues were immediately treated with freshly prepared ice‐cold homogenization buffer (20 mM HEPES, 2 mM EGTA, 0.3 mg/ml dithioerythritol, 0.16 mg/ml phenylmethylsulfonyl fluoride, and 0.020 mg/ml aprotinin) and homogenized. The homogenate was centrifuged at 17,000  ×  *g* for 5 min to obtain the cytoplasmic fraction. The pellet was washed with buffer B (150 mM NaCl; 10 mM HEPES; 1 mM EDTA), centrifuged at 17,000  ×  *g* for 1 min at 4 °C, resuspended in buffer C (25% v/v glycerol; 20 mM HEPES; 400 mM NaCl; 1.2 mM MgCl_2_; 0.2 mM EDTA), vortexed for 30 s and incubated on ice for 10 min (five times) to finally centrifuge at 17,000  ×  *g* for 20 min to obtain the nuclear fraction (Caviedes et al., 2021). RNA expression of GAPDH, U6, miRNA‐384‐3p, and Aak1 in the nuclear and cytoplasmic fractions was detected by RT–qPCR as mentioned above.

### 2.6 Hematoxylin and eosin staining

Rats were euthanized under the anesthesia of pentobarbital sodium (80 mg/kg), and then the hippocampal tissues were removed. Tissues were fixed with 4% paraformaldehyde for 24 h and paraffin embedded. Sections of 4 μM were cut, and staining was carried out according to the hematoxylin and eosin (HE) protocol. Neural injury scoring was performed according to the following standard: no nerve cell death, 0 points; scattered single nerve cell death, 1 point; slight nerve cell death, 2 points; mass nerve cell death, 3 points; and almost complete nerve cell death, 4 points.

### 2.7 Nissl staining

Paraffin sections of hippocampal tissues were deparaffinized and stained with cresyl violet solution for 45 min at 37°C. Next, sections were washed with distilled water and differentiated with gradient concentration ethanol. The differentiation was stopped when the tissue was clear by transferring the sections to distilled water. Then, the sections were dehydrated through a gradient concentration of ethanol and covered with neutral resin. Optical microscopy (Nikon, Tokyo, Japan) was used to observe the neurons in the hippocampal CA1 regions. The number of Nissl bodies was analyzed in a double‐blinded manner with Image‐Pro Plus 6.0.

### 2.8 Cell apoptosis

The cell apoptosis ratio was measured in transfected neurons and hippocampal tissues by using the In Situ Cell Death Detection kit (Roche, Basel, Switzerland). After staining, the positive neurons were randomly observed by a fluorescence microscope (Nikon, Tokyo, Japan) in five fields. The apoptosis ratio was measured by TUNEL‐positive neurons/DAPI‐positive neurons.

### 2.9 Western blot analysis

Protein was extracted from hippocampal tissue or transfected neurons using RIPA lysis buffer containing a protease inhibitor (Promega Corporation, WI, USA). The protein samples were fractionated by SDS–PAGE and transferred to a polyvinylidene difluoride membrane (PVDF, Millipore, MA, USA). Afterwards, the membranes were incubated with 5% nonfat milk for 2 h at room temperature. Then, the membranes were incubated with the primary antibody overnight at 4°C. Then, the membranes were incubated for 2 h with the secondary antibody at room temperature and visualized with a chemiluminescence kit (Vazyme, Nanjing, China). ImageJ software was used to analyze the protein expression. In this study, antibodies against Bax, Bcl‐2, cleaved caspase‐3, PCNA, and Aak1 were diluted to 1:1000 for use, cleaved caspase‐9 was diluted to 1:200, and Ki‐67 was diluted to 1:100. β‐actin was used as the internal control, and the antibody was diluted to 1:5000. The goat anti‐rabbit HRP antibody was used as a secondary antibody and diluted to 1:5000 for use. All antibodies were purchased from Abcam (London, England).

### 2.10 Morris water maze test

The Morris water maze (MWM) test was used to evaluate the learning and memory abilities of rats at the age of 2 months. The MWM consisted of a pool (100 cm × 100 cm × 60 cm) and a platform (1 cm × 1 cm). The pool was filled with warm water (25°C) to 1 cm. Rats were randomly placed in the pool and allowed to swim to the platform. The time that the rats spent swimming to a hidden platform was measured at 90 s, and the rats were allowed to rest on the platform for 20 s. The time was recorded as 90 s if the rats did not find the platform within 90 s, and the rats were also placed on the platform for 20 s to rest. In the acquisition phase, five training sessions were conducted every day for 5 continuous days. After the training, probe trials were performed. The time of plateau quadrant residence and the number of traversing platforms were recorded by computerized tracking/analyzing video systems to suggest the spatial memory and learning ability of the rats.

### 2.11 Dual‐luciferase reporter assay

Aak1 wild type (Aak1 WT) containing the miRNA‐384‐3p binding sites in the 3′UTR of Aak1 was inserted into the firefly luciferase vector. To confirm specific binding, an Aak1 mutant (Aak1 Mut) containing the mutated binding sites of miRNA‐384‐3p in the Aak1 3′UTR was constructed. For the luciferase reporter assay, hippocampal neurons were cultured and plated in 24‐well plates. Each well was transfected with 1 μg Aak1 WT vector or Aak1 Mut vector, 1 μg Renilla luciferase plasmid, and 100 pM miRNA‐384‐3p mimics or NC mimics by using Lipofectamine 3000 (Invitrogen, CA, USA). After 48 h of transfection, the dual‐luciferase reporter assay system (Promega Corporation, WI, USA) was used to measure the firefly and Renilla luciferase activities.

### 2.12 Cell viability assay

Cell viability was detected by the cell counting kit‐8 (CCK8) assay. Transfected hippocampal neurons were seeded onto 96‐well plates at approximately 103 cells/well (100 μl/well). Then, the neurons were cultured for 1 h and mixed with 10 μl CCK8 reagent (Dojindo, Kumamoto, Japan) for 2 h. Next, the optical density was measured at 450 nm by utilizing a Bio‐EL340 automatic microplate reader (Tek Instruments, Hopkinton, USA).

### 2.13 Statistical analysis

All data are presented as the mean ± standard deviation (SD) of three independent experiments. Unpaired Student's *t* test and one‐way ANOVA were used to test the mean difference between groups. Statistical analysis was carried out using GraphPad Prism 7 (GraphPad Inc., San Diego, CA, USA). A *p‐*value < .05 was considered statistically significant.

## 3 RESULTS

### 3.1 Sevoflurane reduces the expression of miRNA‐384‐3p in the rat hippocampus

**FIGURE 1:**
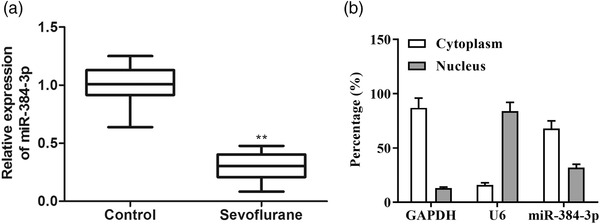
Sevoflurane reduces the expression of miRNA‐384‐3p in the rat hippocampus. (a) The expression of miRNA‐384‐3p was detected by RT–qPCR in the hippocampus of sevoflurane‐exposed rats and control rats. (b) Nuclear and cytoplasmic expression of miRNA‐384‐3p in the hippocampus from control rats was assessed by RT–qPCR. **p < .01, the difference was compared to control rats. The error bars represent the mean ± SD in three independent repetitions

To detect the effect of sevoflurane on miRNA‐384‐3p expression, we collected hippocampal tissues from control and sevoflurane‐exposed neonatal rats and detected the expression of miRNA‐384‐3p through RT–qPCR. The results showed that miRNA‐384‐3p expression was decreased in the hippocampal tissues of sevoflurane‐exposed rats compared with control rats (Figure 1a). miRNA‐384‐3p was primarily located in the cytoplasm in hippocampal tissues (Figure 1b). The results suggested that miRNA‐384‐3p was downregulated by sevoflurane in the rat hippocampus.

### 3.2 miRNA‐384‐3p restores sevoflurane‐induced morphological changes in neurons in the hippocampal CA1 region

**FIGURE 2:**
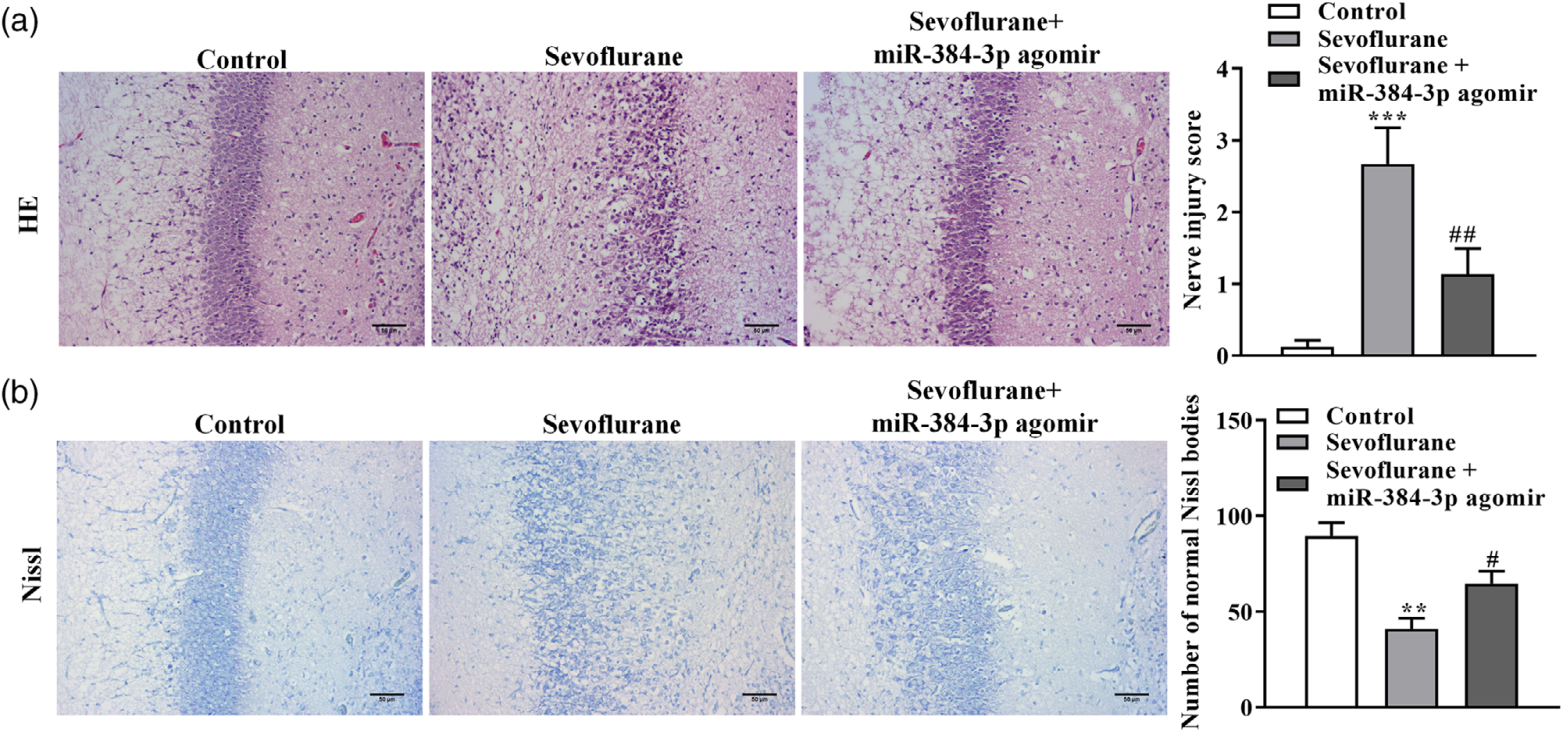
miRNA‐384‐3p restores sevoflurane‐induced morphological changes in neurons in the hippocampal CA1 region. Neonatal rats were exposed to sevoflurane‐induced nerve injury and were divided into two groups; one group was injected with miRNA‐384‐3p agomir into the hippocampus. Normal neonatal rats were used as a negative control. (a) HE staining detected morphological changes in neurons in the hippocampal CA1 region. (b) Nissl staining detected Nissl bodies and neurons in the hippocampal CA1 region. The scale bar is 50 μM. Every experiment had three independent repetitions. ***p < .001, **p < .01 vs. the control group, ^##^ *p* < .01, ^#^ *p* < .05 vs. the sevoflurane group. The error bars represent the mean ± SD in three independent repetitions

Sevoflurane‐induced neurotoxicity has been reported previously (Perez‐Zoghbi et al., 2017). To confirm the role of miRNA‐384‐3p in sevoflurane‐induced neurotoxicity, miRNA‐384‐3p agomir was injected into the rat hippocampus after the first day of sevoflurane exposure. We detected morphological changes in neurons through HE and Nissl staining. The HE results showed that sevoflurane induced neuronal injury and decreased the number of neurons in the hippocampal CA1 regions, and the decreased injury and number of neurons were attenuated by the miRNA‐384‐3p agomir (Figure 2a). The Nissl results showed that Nissl bodies and neurons were decreased in the hippocampal CA1 regions of sevoflurane‐exposed rats compared with control rats. The sevoflurane‐induced decrease in Nissl bodies was attenuated by the miRNA‐384‐3p agomir (Figure 2b). These results demonstrated that miRNA‐384‐3p restored the sevoflurane‐induced morphological changes in neurons in the hippocampal CA1 regions.

### 3.3 miRNA‐384‐3p inhibits sevoflurane‐induced neuronal apoptosis in the hippocampal CA1 region

**FIGURE 3:**
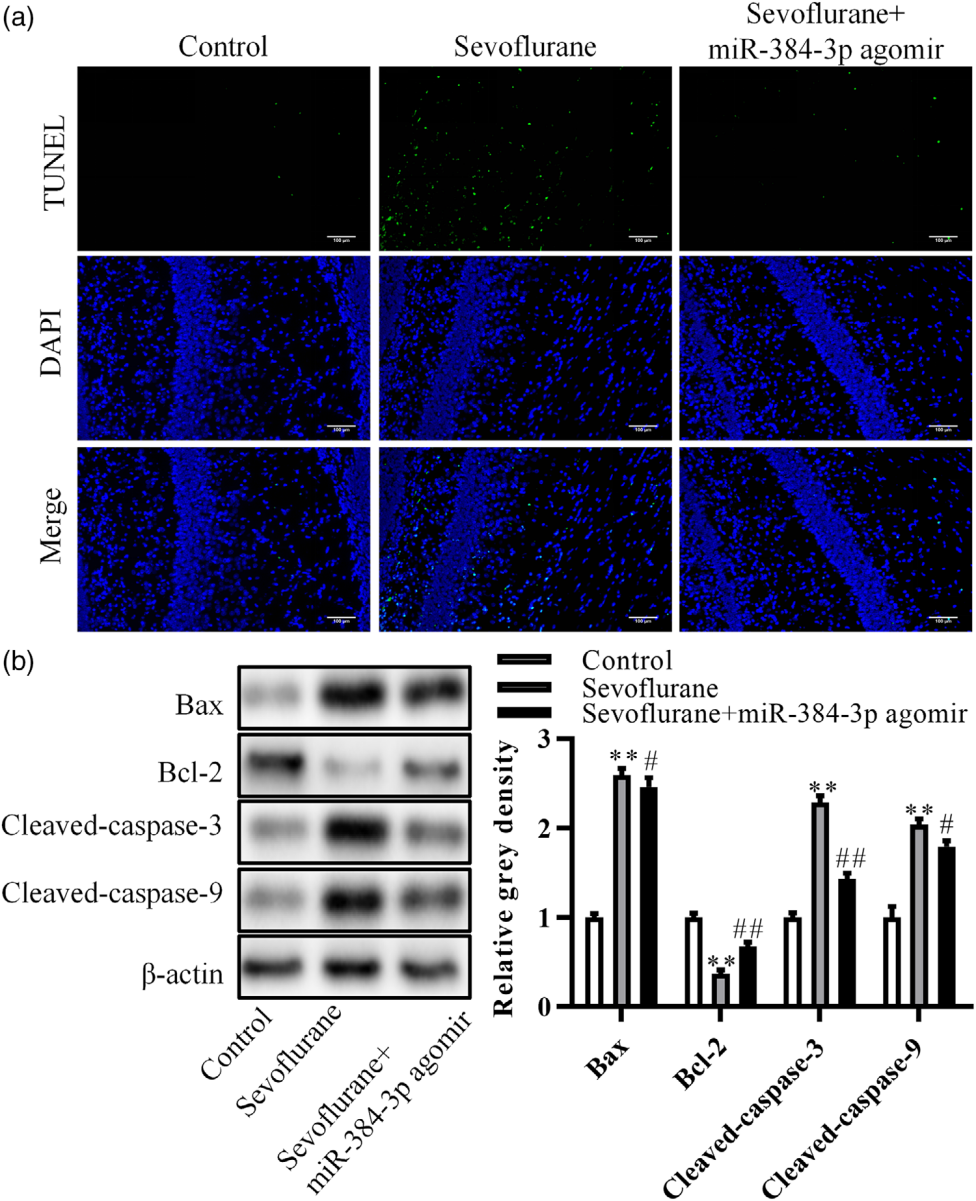
miRNA‐384‐3p inhibits sevoflurane‐induced neuronal apoptosis in the hippocampal CA1 region. Neonatal rats were exposed to sevoflurane‐induced nerve injury and were divided into two groups; one group was injected with miRNA‐384‐3p agomir into the hippocampus. Normal neonatal rats were used as a negative control. (a) Cell apoptosis was detected by a TUNEL assay in the hippocampal CA1 region. (b) Western blot analysis of the expression of apoptosis‐related genes. **p < .01. The difference was compared to control rats. ##*p* < .01, #*p* < .05, the difference was compared to sevoflurane‐treated rats. The error bars represent the mean ± SD in three independent repetitions

We detected the function of miRNA‐384‐3p in sevoflurane‐induced neuronal apoptosis through the TUNEL assay and Western blot assay. The TUNEL assay results demonstrated that the apoptosis ratio of neurons was increased in the hippocampal CA1 region of sevoflurane‐treated rats compared with control rats. Overexpression of miRNA‐384‐3p inhibited the apoptosis induced by sevoflurane (Figure 3a). Similar to the TUNEL assay results, Western blot results showed that the expression of Bax, cleaved‐caspase‐3, and cleaved‐caspase‐9 was increased. Meanwhile, Bcl‐2 expression was decreased in the hippocampal CA1 region of sevoflurane‐treated rats compared with control rats. Overexpression of miRNA‐384‐3p attenuated sevoflurane‐induced expression changes in these apoptosis‐related genes (Figure 3b). These results suggested that miRNA‐384‐3p inhibited sevoflurane‐induced neuronal apoptosis in the hippocampal CA1 region.

### 3.4 miRNA‐384‐3p improves the spatial memory and learning ability of sevoflurane‐treated rats

**FIGURE 4:**
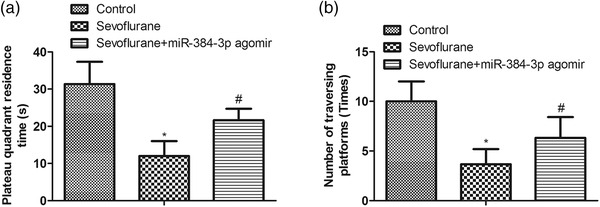
miRNA‐384‐3p improves the spatial memory and learning ability of sevoflurane‐treated rats. Neonatal rats were exposed to sevoflurane‐induced nerve injury and were divided into two groups; one group was injected with miRNA‐384‐3p agomir into the hippocampus. Normal rats were used as a negative control. When rats were at the age of 2 months, plateau quadrant residence (a) and the number of traversing platforms (b) were detected by the MWM test. *p < .05 vs. the control group, ^#^ *p* < .05 vs. the sevoflurane group. The error bars represent the mean ± SD. Every experiment had three independent repetitions

Next, we tested the function of miRNA‐384‐3p in sevoflurane‐induced changes in spatial memory and learning ability through the MWM test. The results showed that the time of plateau quadrant residence and the number of traversing platforms were reduced in sevoflurane‐treated rats compared with control rats, suggesting that sevoflurane impaired the spatial memory and learning ability of rats (Figure 4a,b). Meanwhile, overexpression of miRNA‐384‐3p increased the plateau quadrant residence time and the number of traversing platforms in sevoflurane‐treated rats, suggesting that miRNA‐384‐3p attenuated sevoflurane‐induced injury to spatial memory and learning ability (Figure 4a,b). These results demonstrated that miRNA‐384‐3p had a protective effect on spatial memory and learning ability in rats.

### 3.5 Aak1 is a target gene of miRNA‐384‐3p

**FIGURE 5:**
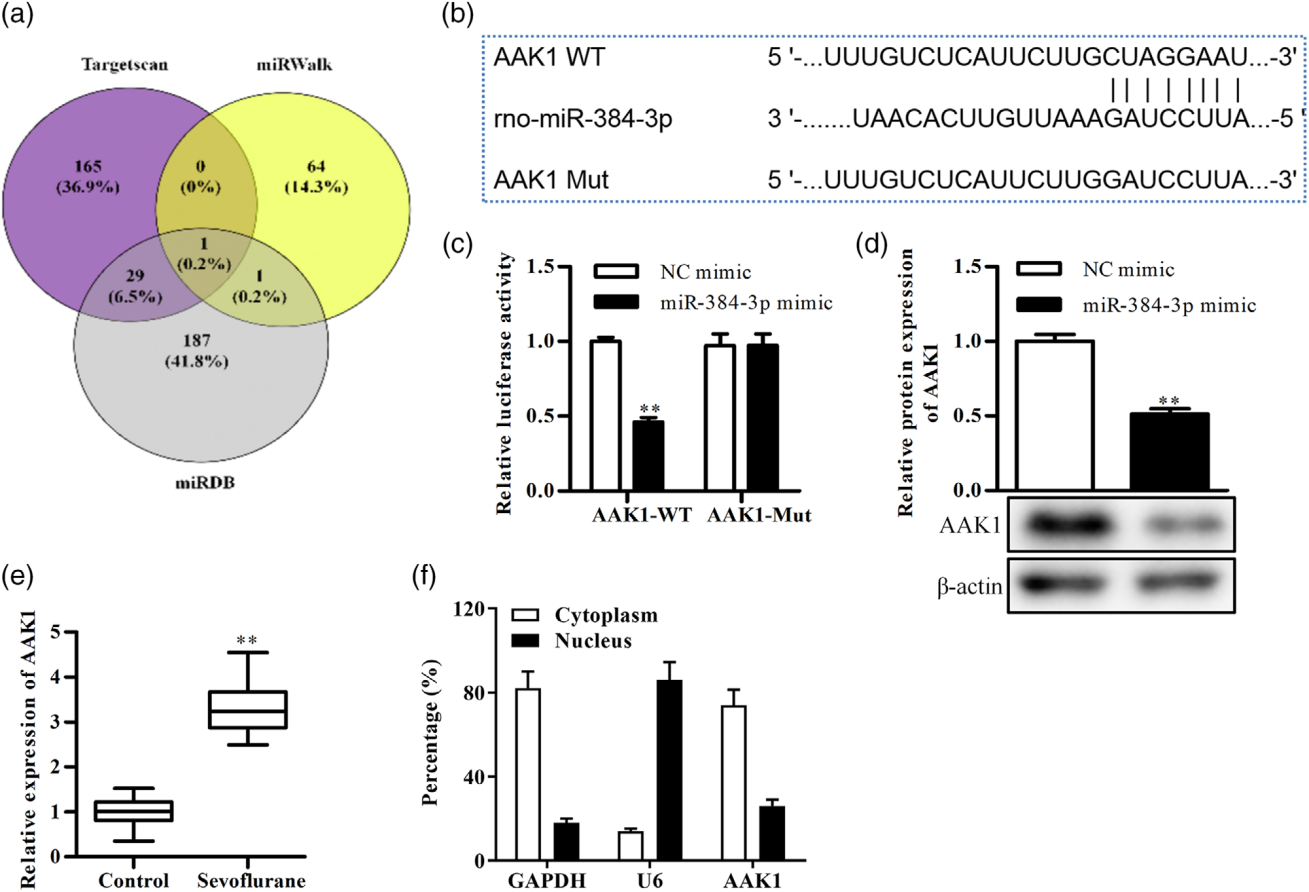
Aak1 is a target gene of miRNA‐384‐3p. (a) Prediction of target genes of miRNA‐384‐3p through the miRDB, miRWalk, and TargetScan databases. (b) The putative target sequence of miRNA‐384‐3p in the 3′UTR of Aak1 and the mutated sequence. (c) Luciferase assays in neurons transfected with Aak1 WT or Aak1 Mut and NC mimics or miR‐384 mimics. (d) Western blot analysis of Aak1 expression in neurons transfected with miRNA‐384‐3p mimics or NC mimics. (e) RT‐qPCR detected Aak1 expression in the hippocampus of sevoflurane‐treated rats and control rats. (f) Nuclear and cytoplasmic expression of Aak1 in the hippocampus from normal rats was assessed by RT‐qPCR. **p < .01. The difference was compared to control rats or transfected NC mimic neurons. The error bars represent the mean ± SD in three independent repetitions

To explore the underlying mechanism of miRNA‐384‐3p in sevoflurane‐induced nerve injury, we used the TargetScan, miRWalk, and miRDB databases to predict the target genes of miRNA‐384‐3p. The predicted results showed that Aak1 was the only target gene of miRNA‐384‐3p in the three databases (Figure 5a). The predicted binding sequence of Aak1 and miRNA‐384‐3p is shown in Figure 5b. The luciferase assay was used to confirm the binding site, and the results showed that the luciferase activity was decreased in Aak1 3′UTR WT and miRNA‐384‐3p mimic cotransfected neurons compared with Aak1 3′UTR WT and NC mimic cotransfected neurons. However, the luciferase activity was not significantly changed in Aak1 3′UTR MUT‐transfected neurons (Figure 5c). To confirm that miRNA‐384‐3p regulates Aak1 expression, Western blotting was performed on neurons transfected with NC mimics or miRNA‐384‐3p mimics. The results showed that the expression of Aak1 was decreased in miRNA‐384‐3p mimic‐transfected neurons compared with NC mimic‐transfected neurons (Figure 5d). Additionally, RT‐qPCR results showed that Aak1 was upregulated in the hippocampus of sevoflurane‐treated rats compared with control rats (Figure 5e). Moreover, Aak1 was primarily located in the cytoplasmic fraction in the hippocampus of control rats (Figure 5f). These results demonstrated that Aak1 was a target gene of miRNA‐384‐3p and that its expression was negatively regulated by miRNA‐384‐3p.

### 3.6 miRNA‐384‐3p alleviates sevoflurane‐induced neuronal apoptosis and nerve injury through Aak1

**FIGURE 6:**
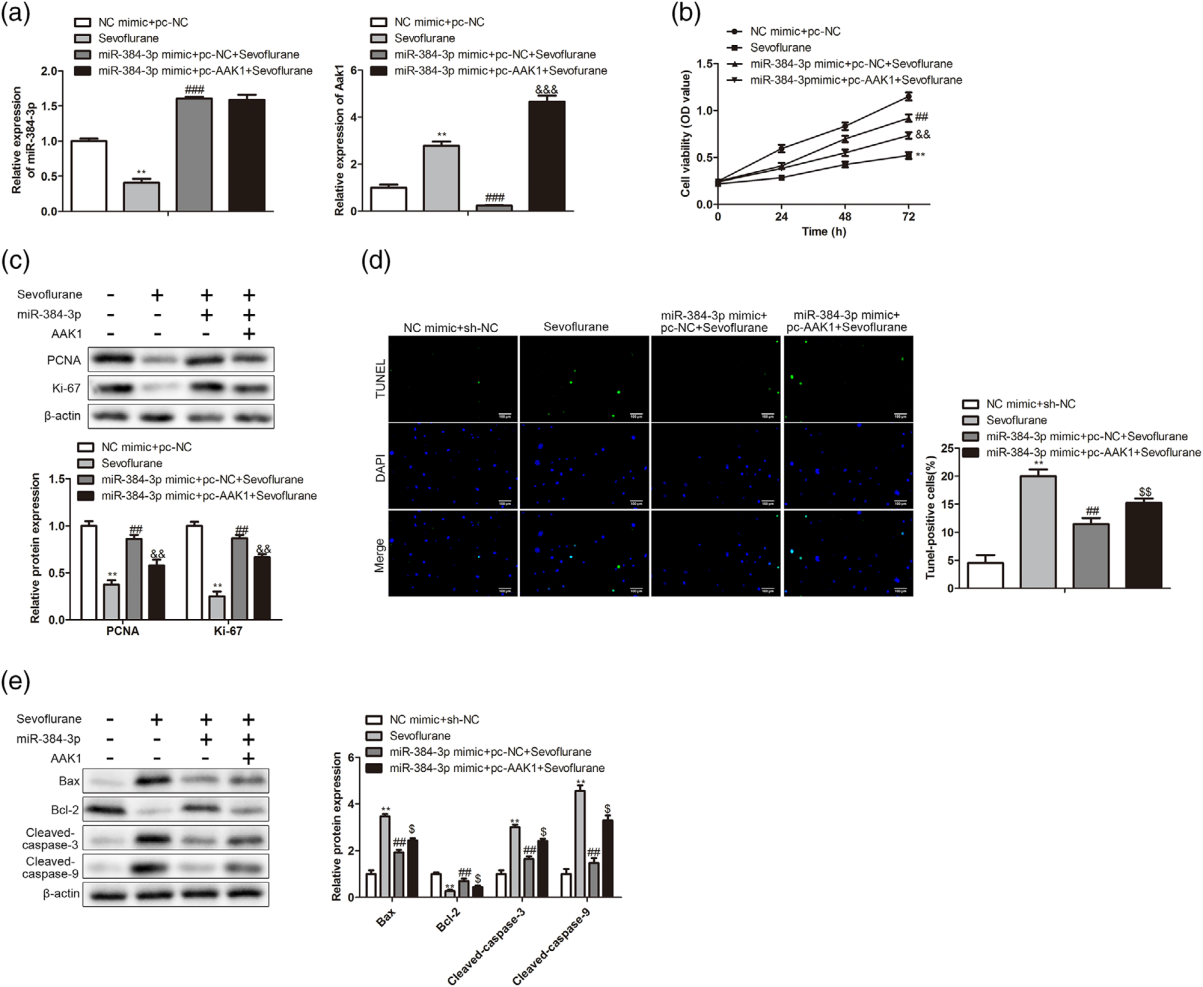
miRNA‐384‐3p alleviates sevoflurane‐induced neuronal apoptosis and nerve injury through Aak1. Hippocampal neurons were divided into four groups, including NC mimic‐ and pc‐NC‐transfected neurons cultured under control conditions, NC mimic‐ and pc‐NC‐transfected neurons cultured with 1% sevoflurane, miRNA‐384‐3p mimic‐ and pc‐NC‐transfected neurons cultured with sevoflurane, miRNA‐384‐3p mimic‐, and pc‐Aak1‐transfected neurons cultured with sevoflurane. (a) RT‐qPCR detected miRNA‐384‐3p and Aak1 levels. (b) CCK8 assay detected cell viability. (c) Western blot analysis of the expression of proliferation‐related genes. (d) TUNEL assay detected cell apoptosis. (e) Western blot analysis of apoptosis‐related gene levels. **p < .01 vs. the NC mimics + pc‐NC group. ###*p *< .001, ##*p *< .01 vs. the sevoflurane group. &&&*p *< .001, &&*p *< .01, &*p *< .05 vs. the miR‐384‐3p mimic + pc‐NC + sevoflurane group. The error bars represent the mean ± SD in three independent repetitions

To confirm whether miRNA‐384‐3p plays a role in sevoflurane‐induced nerve injury through Aak1, we transfected miRNA‐384‐3p mimics and the Aak1 vector into hippocampal neurons simultaneously, and the neurons were cultured with sevoflurane. The RT‐qPCR results showed that miRNA‐384‐3p expression was decreased in sevoflurane‐treated neurons compared to control neurons, and miRNA‐384‐3p mimics restored the expression of miRNA‐384‐3p. Meanwhile, the expression of Aak1 was increased in sevoflurane‐treated neurons compared with control neurons. miRNA‐384‐3p mimics decreased the expression of Aak1 in sevoflurane‐treated neurons, and the miRNA‐384‐3p‐induced decrease in Aak1 was partially restored by transfection with the Aak1 vector (Figure 6a). To detect whether Aak1 participated in the regulation of miRNA‐384‐3p on sevoflurane‐induced cell viability, a CCK8 assay was used. The results showed that miRNA‐384‐3p attenuated the inhibitory effect of sevoflurane on cell viability, while the miRNA‐384‐3p effect was remarkably undermined after the overexpression of Aak1 (Figure 6b). Western blotting was used to measure proliferation‐related gene expression at the protein level. The results showed that sevoflurane inhibited the expression of PCNA and Ki‐67, which was partially restored by miRNA‐384‐3p. Meanwhile, overexpression of Aak1 attenuated miRNA‐384‐3p‐mediated expression changes in PCNA and Ki‐67 in sevoflurane‐treated neurons (Figure 6c). The TUNEL assay was used to detect whether Aak1 participated in the regulation of miRNA‐384‐3p on sevoflurane‐induced cell apoptosis, and the results showed that overexpression of miRNA‐384‐3p reduced the apoptosis of hippocampal neurons induced by sevoflurane, while the miRNA‐384‐3p effect was inhibited by increasing the expression of Aak1 (Figure 6d). Similar to the results, the Western blot results demonstrated that sevoflurane‐induced changes in apoptosis‐related genes were attenuated by miRNA‐384‐3p. Meanwhile, Aak1 overexpression restored the miRNA‐384‐3p‐mediated changes in the expression of these genes in sevoflurane‐treated hippocampal neurons (Figure 6e). These results demonstrated that miRNA‐384‐3p alleviated sevoflurane‐induced neuronal apoptosis and nerve injury through Aak1.

## 4 DISCUSSION

Anesthesia is widely used in modern medicine; however, a multitude of evidence has demonstrated that anesthesia increases the risk of neurodevelopmental defects (Warner et al., 2018; Zhang et al., 2016). For instance, ropivacaine exposure induces significant sciatic nerve injury in diabetic rats (Yu et al., 2019). Ketamine, midazolam, or a combination of the two drugs induce apoptotic neurodegeneration in the developing mouse brain (Young et al., 2005). Therefore, exploring the methods of reducing the injury induced by anesthesia is important and necessary. Sevoflurane is an anesthetic and contributes to neurological disorders and neurodegeneration in the development of the brain and affects memory and cognition (O'Farrell et al., 2018; Zhang et al., 2016). Sevoflurane at subanesthetic concentrations triggers neuronal apoptosis in 7‐day‐old mouse brains (Zhang et al., 2008). Sevoflurane exposure repeatedly induces cognition‐related biochemical changes in the hippocampus and impairs learning and memory ability (Guo et al., 2018). Therefore, we established a sevoflurane neurotoxicity model in neonatal rats through repeated exposure to sevoflurane.

Previous studies have reported that neurotoxicity induced by anesthesia is regulated by miRNA (Bahmad et al., 2020). For example, miR‐34a expression is upregulated in propofol‐treated neurons and rats. Meanwhile, inhibition of miR‐34a improves propofol‐induced cognitive dysfunction by suppressing cell apoptosis and recovering the expression of MAPK/ERK pathway genes (Xin, 2018). miR‐124 increases ketamine‐induced apoptosis in the hippocampal CA1 region and improves the memory performance of mice (H. Xu et al., 2015). miR‐384 is also involved in the progression of brain development, cognition, and pathophysiology of neurological disorders (Gu et al., 2015). However, the roles of miRNA‐384‐3p in anesthesia‐induced neurotoxicity remain unclear. Here, we detected the expression of miRNA‐384‐3p in sevoflurane‐exposed rat hippocampi and found that sevoflurane decreased the expression of miRNA‐384‐3p. A miRNA‐384‐3p agomir was injected into neonatal rats to overexpress miRNA‐384‐3p. We further confirmed that miRNA‐384‐3p improved neuronal morphology, neuronal apoptosis, and learning and memory ability in sevoflurane‐treated rats.

miRNAs mainly regulate the mRNA degradation or posttranscriptional repression of the targeted gene (Saliminejad et al., 2019). To explore the mechanism of miRNA‐384‐3p in sevoflurane‐induced nerve injury, we predicted and confirmed that Aak1 is a target gene of miRNA‐384‐3p. Aak1 plays vital roles in neuropathic pain, schizophrenia, Parkinson's disease and other neuropathic disorders (Abdel‐Magid, 2017). For example, Fu et al. found that Aak1 expression is highest on day 14 and is reduced on day 30 in the Aβ_1‐42_‐induced AD model. The expression of Aak1 is negatively correlated with cognitive ability by regulating the process of clathrin‐mediated endocytosis (Fu et al., 2018). Kostich, Walter et al. discovered that Aak1 knockout mice have an antinociceptive phenotype, which may be a novel therapeutic approach for neuropathic pain by inhibiting Aak1 kinase (Kostich et al., 2016). Leger, Helene et al. found that Ndr kinases inhibit the proliferation of terminally differentiated cells and modulate the function of interneuron synapses through Aak1 (Leger et al., 2018). However, the role of Aak1 in anesthesia‐mediated nerve injury remains unknown. Here, we confirmed that Aak1 expression was negatively regulated by miRNA‐384‐3p in hippocampal neurons. Meanwhile, we demonstrated that miRNA‐384‐3p alleviated sevoflurane‐induced neuronal apoptosis and nerve injury by inhibiting the expression of Aak1 via rescue experiments.

## 5 CONCLUSION

In neonatal rats, we confirmed the roles and mechanisms of miRNA‐384‐3p in sevoflurane‐induced nerve injury, including hippocampal neuron apoptosis and memory impairment. The findings of our study suggest that miRNA‐384‐3p could be a promising strategy for reducing sevoflurane‐induced nerve injury in clinical surgery.

## CONFLICT OF INTEREST

The authors declare that they have no conflict of interest.

## AUTHOR CONTRIBUTIONS

Xuan Gao and Hao Pei conceived and designed the study. Xuan Gao and Yuanyuan Chen performed the literature search and data extraction. Hao Pei drafted the manuscript. All authors read and approved the final version of the manuscript.

### PEER REVIEW

The peer review history for this article is available at https://publons.com/publon/10.1002/brb3.2556

## Data Availability

All data generated or analyzed during this study are included in the article.
